# Intermittent Overconsumption of High Fat Diet Promotes Microglial Reactivity in the Hypothalamus and Hindbrain of Female Rats

**DOI:** 10.3390/cells14030233

**Published:** 2025-02-06

**Authors:** Alexis A. Campanile, Lisa A. Eckel

**Affiliations:** 1 Program in Neuroscience, Department of Psychology, Florida State University, Tallahassee, FL 32304, USA; eckel@psy.fsu.edu; 2 Division of Pharmacology and Toxicology, College of Pharmacy, University of Texas at Austin, Austin, TX 78712, USA

**Keywords:** binge eating, high fat diet, hypothalamus, arcuate nucleus, obesity, inflammation, microglial reactivity

## Abstract

Elevated proinflammatory cytokines were reported in binge eating spectrum disorders characterized by intermittent overconsumption during periods of otherwise normal or restricted food intake. It is unknown whether binge eating promotes neuroinflammation, similar to that observed following chronic overconsumption of a high fat diet (HFD) in rodents. Here, we used a rodent model of binge-like eating to test the hypothesis that intermittent overconsumption of HFD promotes microglial reactivity in brain areas that control food intake. To promote overconsumption, one group of rats received chow plus intermittent access to HFD (INT). Control groups received either chow only (CHOW) or chow plus continuous access to HFD (CONT). Following behavioral testing, brains were processed to visualize ionized calcium-binding adaptor molecule 1 (Iba1), a microglial marker. INT rats consumed more calories than the control rats on days when the HFD was available, and fewer calories than the control rats on days when they only had access to chow. Despite consuming fewer total calories and 50% fewer fat calories, lean INT rats developed a pattern of microglial reactivity in feeding-relevant brain areas similar to obese CONT rats. We conclude that intermittent overconsumption of HFD, without diet-induced weight gain, promotes microglial reactivity in brain regions that control feeding.

## 1. Introduction

Binge eating is a form of dysregulated eating behavior that occurs during periods of otherwise normal or restricted food intake. Episodes of binge eating involve the consumption of an objectively large amount of food in a discrete period of time that is accompanied by a loss of control over eating [1]. Because binge eating is a core feature of multiple binge eating spectrum disorders, including binge eating disorder (BED), bulimia nervosa (BN), and binge–purge anorexia nervosa, a greater understanding of the biological causes and consequences of binge eating could advance the prevention and treatment of multiple eating disorders.

Chronic disease states that are often comorbid with binge eating spectrum disorders, including obesity and depression, are associated with low-grade, systemic inflammation [2,3]. This has led researchers to investigate whether inflammation or altered inflammatory processes may contribute to the onset or maintenance of binge eating spectrum disorders. An association between inflammatory disease and eating disorder risk was first reported in a population-based Finnish study [4]. This finding was extended in a Danish study, which found that familial autoimmune disease predicted eating disorder risk in offspring, and having an autoinflammatory or autoimmune disease in childhood increased the risk of developing BN by 73% [5]. More recently, the Avon Longitudinal Study of Parents and Children found that those who later developed BED or BN had a higher body mass index and poorer metabolic and inflammatory profiles at age 6 relative to children who did not develop an eating disorder [6]. Taken together, these studies suggest that disorders of the immune system may play a causal role in the development of binge eating spectrum disorders.

Like obesity, binge eating spectrum disorders may be associated with chronic, low-grade inflammation as some studies report elevated plasma levels of inflammatory cytokines in individuals with BN and BED compared to healthy controls [7,8,9,10,11]. Moreover, our group recently reported an increase in plasma interleukin-6 levels in individuals with binge eating spectrum disorders that was not accounted for by adiposity, body mass index, or depression [12]. Associations between other markers of inflammation and binge eating spectrum disorders were also reported. For example, individuals with BED had a poorer inflammatory profile, characterized by elevated C-reactive protein and white blood cell counts, and lower levels of adiponectin, which supports insulin sensitivity and has anti-inflammatory effects, in comparison to individuals with a similarly elevated body mass index but not diagnosed with BED [13,14]. These studies are particularly interesting as they suggest that binge eating, rather than the weight gain associated with BED, may be uniquely associated with inflammation.

While the deleterious effects of chronic high fat diet (HFD) consumption are well characterized, much less is known about the effects of intermittently overconsuming HFD during episodes of binge eating. In rodent models of diet-induced obesity, chronic overconsumption of HFD promotes a sustained inflammatory response in the brain [15,16]. This neuroinflammation, which is particularly pronounced in the arcuate nucleus of the hypothalamus (ARC) [16,17,18,19], is mediated primarily by microglia, the resident immune cells in the brain [19]. Microglia respond to high levels of saturated fatty acids by increasing production of inflammatory cytokines and activating inflammatory signaling pathways [16]. While the microglial response to an acute neural insult can be adaptive, a more prolonged response, like that induced by chronic HFD consumption, could promote synaptic remodeling, cellular stress, and apoptosis [20,21,22,23]. As such, persistent microglial reactivity may alter brain structure and function in ways that could promote or exacerbate disease states, including obesity and binge eating spectrum disorders.

The current study used a rodent model of binge eating to investigate whether intermittent overconsumption of HFD promotes a neuroinflammatory response, characterized by microglial reactivity. It should be noted that modeling eating disorders, including binge eating spectrum disorders, is particularly challenging as it is impossible to capture the full spectrum of behavioral, physiological, and psychological symptoms in a single paradigm in rodents. However, given the tight conservation of feeding circuits across species, it is possible to recapitulate binge eating, a core feature of all binge eating spectrum disorders, in rodents. As such, rodent models of binge eating offer a unique opportunity to study the biological mechanisms underlying the dysregulated homeostatic and hedonic eating behavior that occurs in binge eating spectrum disorders. The model used here—the intermittent HFD model—has good face validity in modeling binge eating as it does not rely upon food restriction or aversive stressors to promote overconsumption. In this model, freely fed rodents are maintained on a chow diet and given 22 h access to HFD at 4-day intervals. On days when HFD is available, rodents consume large amounts of HFD, eating more than three times that of the chow-fed control rats [24]. Here, we used this model to investigate whether intermittent overconsumption of HFD induces microglial reactivity in brain areas that control meal size, including the ARC and nucleus of the solitary tract (NTS) [23,25,26,27]. Female rats were used because binge eating spectrum disorders disproportionately affect women. Following three cycles of intermittent HFD access, brain tissue was collected and processed for the expression of ionized calcium-binding adaptor molecule 1 (Iba1), a cytoplasmic protein marker of microglial cells [28]. We hypothesized that the intermittent overconsumption of HFD promotes microglial reactivity in the ARC and NTS of female rats.

## 2. Materials and Methods

### 2.1. Animals, Housing, and Diets

Naïve, female Sprague Dawley rats (Charles River Breeding Laboratory, Raleigh, NC, USA; 12 weeks, 200–230 g), were individually housed in polycarbonate cages in a temperature-controlled room on a 12:12 h light/dark cycle. Throughout the study, all rats had ad lib access to water and chow (5001 Rodent Diet, LabDiet; St. Louis, MO, USA; 13% fat; 3.34 kcal/g). As described in greater detail below, groups of chow-fed rats were also given either intermittent or continuous access to 45% HFD (D12451, Research Diets, New Brunswick, NJ, USA; 45% fat; 4.73 kcal/g). Animal procedures were approved by the Institutional Animal Care and Use Committee and conformed to the National Institutes of Health Guide for the Care and Use of Animals [29]. Data reporting followed Animal Research: Reporting of In Vivo Experiments Guidelines 2.0 [30].

### 2.2. Intermittent HFD Paradigm

At study onset, rats were assigned to one of three groups, matched for baseline levels of food intake and body weight: chow diet (CHOW, *n* = 12), continuous HFD (CONT, *n* = 13), or intermittent HFD (INT, *n* = 15). To establish a pattern of recurrent overconsumption, INT rats were given 48 h access to HFD on days 1 and 2 (HFD adaptation phase) and then given 22 h access to HFD at 4-day intervals on days 6, 10, and 14 (intermittent binge days) in addition to having ad lib daily access to their maintenance (chow) diet. This diet choice on binge days allowed us to examine the face validity of this rodent model as individuals with binge eating spectrum disorders typically consume highly palatable, energy dense foods during episodes of binge eating behavior. During this same 14-day period, CONT rats were given *ad lib* access to both chow and HFD and CHOW rats were given access to chow only. The diets were presented in stainless-steel hoppers placed on cage lids. Food intake and body weight were measured daily. On “binge” days, food intake was recorded at 30 min and 2 h following chow/HFD access in all diet groups. Food intake (g) was converted to kilocalories (kcal) to compare total caloric intake across diet groups. On the final day, animals were anesthetized with an intraperitoneal injection of Euthanasia III Solution (100 mg/kg, Covetrus, Portland, ME, USA) and then perfused transcardially with 0.15 M saline followed by 4% paraformaldehyde (Sigma, Carlsbad, CA, USA). Brains were dissected, post-fixed overnight, and cryoprotected in sucrose prior to collecting coronal sections (35 μm) through the ARC and NTS on a freezing-sliding microtome.

### 2.3. Immunohistochemistry

Using our previously described approach [18], free-floating brain sections were washed in phosphate buffer (P3619, Sigma, 0.1 M) and then incubated in 0.5% sodium borohydride (P2256, Sigma) in phosphate buffer for 20 min. Following four washes in phosphate buffer, the tissue was immersed in 0.3% triton-X (X-100, Sigma) in phosphate buffer for 30 min. Tissue sections were then incubated overnight (22 h) in Iba1 primary antibody (019-19741, Wako Chemicals (Osaka, Japan), 1:50,000). The following day, the tissue was rinsed four times in phosphate buffer and then incubated for 1 h in biotinylated secondary antibody (BA-1000, Jackson Immuno-Research (West Grove, PA, USA), 1:500). Following three rinses in phosphate buffer, the tissue was immersed in an avidin-biotin complex (PK-6100, Vector Labs, Newark, CA, USA) for 90 min. To visualize Iba1 immunoreactivity, tissue sections were rinsed in phosphate buffer and then stained in diaminobenzidine tetrahydrochloride for 11 min (SK-4100, Vector Labs), after which they were washed twice in deionized water. Processed tissue sections were mounted on glass slides and coverslipped with permount (SP 15-500, Fisher, Waltham, MA, USA).

### 2.4. Iba1 Quantification

Images of Iba1-processed tissue were captured (blinded to the diet group) at 20× magnification using an Olympus AX70 microscope equipped with a digital camera (AmScope, Irvine, CA, USA). Iba1-positive cells were quantified within a standardized (200 × 200 μm) region of interest (ROI) within the ARC and NTS as in previous studies [19,31]. The number of Iba1-positive cells, defined by the presence of a distinct soma within the boundary of the ROI, were counted manually. Because reactive microglia display morphological changes, including enlarged cell bodies and retracted and thickened processes [32], we used a skeletonization analysis (FIJI software, Version 2.16.0) to quantify changes in microglial branch number and length as described previously [33]. For each animal, the total number of branches and summed branch length were divided by the number of microglia within each ROI. The ARC was quantified at the level of the median eminence (−2.16 to −3.48 from bregma; *n* = 5–7 sections per rat bilaterally) and the NTS was quantified at the level of the area postrema (−13.68 to −14.40 from bregma; *n* = 4 sections per rat bilaterally). Some animals were excluded from analysis due to tissue damage, including one CONT animal and three INT animals.

### 2.5. Statistical Analysis

Data are presented as means ± standard error of the mean. Group differences in daily food intake, daily body weight, and short-term (30 min and 2 h) food intake on “binge” days were assessed via two-factor analyses of variance tests (ANOVAs) (diet × day). Because the analyses of short-term food intake on days 6, 10, and 14 yielded no main (*F*(2, 74) = 0.004–1.43, n.s.) or interactive (*F*(4, 74) = 0.46–2.11, n.s.) effects of day, short-term food intake on day 14 (chosen as a representative intermittent HFD access day) was reanalyzed via one-way ANOVAs. Group differences in total (14-day) food intake, weight gain, number of Iba1-positive cells, average Iba1 branch number, and average summed Iba1 branch length were assessed via one-way ANOVAs. Differences in daily, 30 min, and 2 h HFD intake between CONT and INT rats were analyzed via independent *t*-tests. Significant (*p* < 0.05) ANOVA effects were examined using Tukey’s post hoc tests. Correlational analyses examined the association between body weight gain and number of Iba1-positive cells in the ARC across treatment groups. The data were analyzed using Prism Version 9.5.1 (GraphPad) statistical software.

## 3. Results

### 3.1. Effects of Intermittent Access to HFD on Food Intake and Body Weight

**Figure 1:**
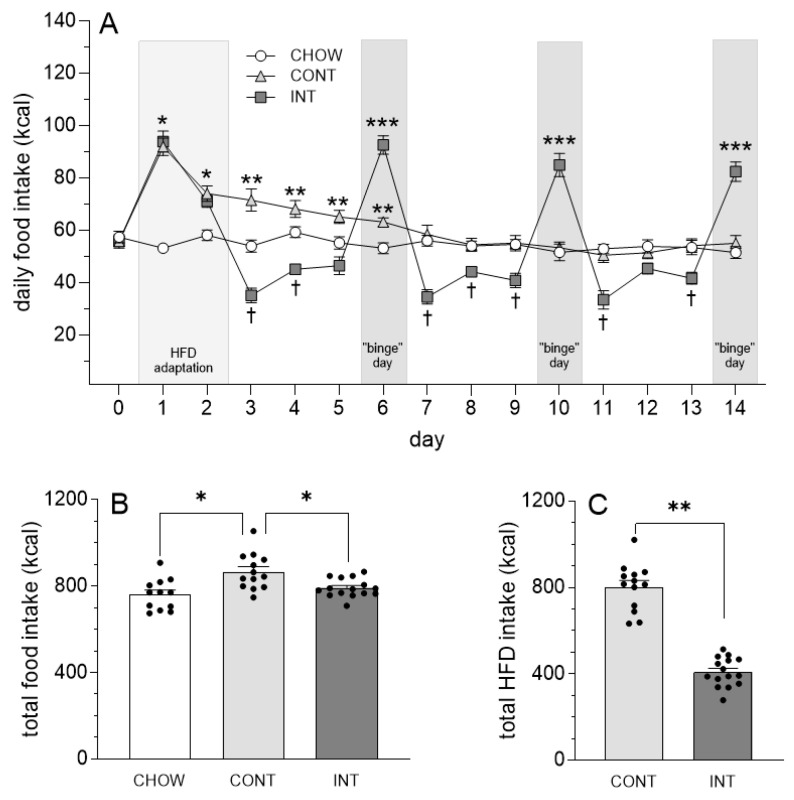
Effects of intermittent access to HFD on daily and cumulative food intake. (**A**) Access to HFD on adaptation days 1 and 2 (denoted by light gray shading) increased caloric intake in CONT and INT rats, relative to CHOW rats (* *p* < 0.0001). Caloric intake remained elevated through day 6 in CONT rats (** *p* < 0.01), whereas INT rats displayed increases in caloric intake only on days when HFD was available (days 6, 10, and 14; denoted by dark gray shading; *** *p* < 0.0001). Following bouts of overconsumption, INT rats displayed a compensatory decrease in caloric intake on days following HFD access († *p* < 0.01). (**B**) Examination of cumulative food intake (chow plus HFD) across the 14-day paradigm revealed that CONT rats consumed more calories than INT and CHOW rats (* *p* < 0.01). (**C**) Examination of cumulative HFD intake also revealed greater caloric intake of HFD in CONT rats, relative to INT rats (** *p* < 0.0001).

Analysis of daily food intake revealed a group by day interaction, *F*(28, 518) = 27.73, *p* < 0.0001, η^2^ = 0.36 (Figure 1A). As expected, caloric intake was increased in the CONT and INT rats, relative to the CHOW rats, during the first two days of HFD adaptation (*p*-values < 0.01). This increase in caloric intake persisted through day 6 in CONT rats (*p*-values < 0.05). In comparison, increases in caloric intake in INT rats were only observed on days when HFD was available (on days 6, 10, and 14). On these days, INT rats consumed more calories than CHOW and CONT rats (*p*-values < 0.0001). INT rats also displayed compensatory decreases in caloric intake on the days following intermittent HFD consumption (days 7 and 11; *p*-values < 0.01).

Group differences in total food intake (chow plus HFD consumed across the 14 days) were also detected, *F*(2, 37) = 8.35, *p* < 0.01, η^2^ = 0.31, with CONT rats consuming more calories than CHOW and INT rats (*p*-values < 0.05) (Figure 1B). No difference in total caloric intake was detected between INT and CHOW rats. Examination of total HFD intake across the 14 days revealed that CONT rats consumed more than twice the number of HFD calories than INT rats, *t*(26) = 11.69, *p* < 0.0001, d = 4.48 (Figure 1C).

**Figure 2:**
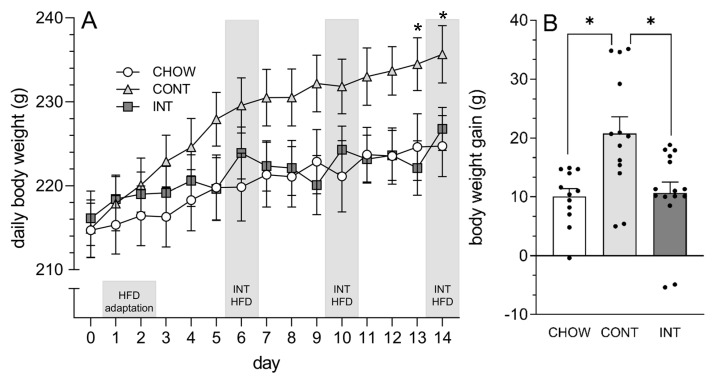
Effects of intermittent access to HFD on daily body weight and cumulative weight gain. (**A**) Daily body weight was increased in CONT rats, relative to CHOW and INT rats, by day 13. There were no significant differences in body weight between INT and CHOW rats on any test day. (**B**) Cumulative (14-day) body weight gain was increased in CONT rats, relative to CHOW and INT rats. There was no difference in weight gain between CHOW and INT rats. * CONT > CHOW and INT, *p* < 0.01.

Daily body weight was influenced by a group × time interaction, *F*(28, 518) = 5.73, *p* < 0.0001, η^2^ = 0.02, with CONT rats weighing more than the CHOW and INT rats by day 13 (*p*-values < 0.05) (Figure 2A). In contrast, no differences in daily body weight were detected between the INT and CHOW rats. Cumulative weight gain across the 14 days was also influenced by diet, *F*(2, 37) = 7.92, *p* < 0.01, η^2^ = 0.30, with CONT rats gaining more weight and thus developing a diet-induced obesity phenotype in comparison to CHOW and INT rats (*p*-values < 0.01) (Figure 2B). No difference in cumulative weight gain was detected between CHOW and INT rats.

### 3.2. Intermittent Access to HFD Promoted Overconsumption

**Figure 3:**
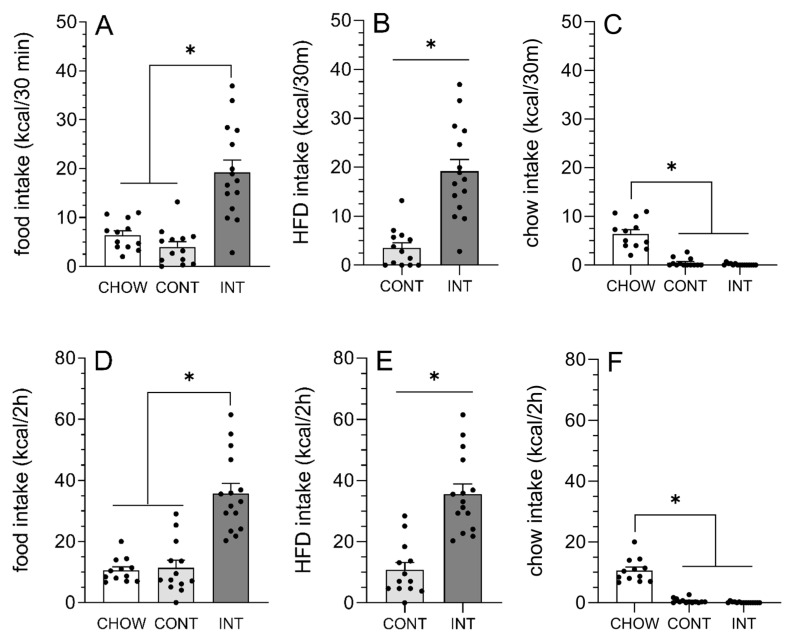
Overconsumption was observed in INT rats, relative to CONT and CHOW rats, at 30 min and 2 h following access to HFD on the final intermittent HFD day (day 14). Total food intake (chow plus HFD) was increased in INT rats, relative to CHOW and CONT rats, at (**A**) 30 min and (**D**) 2 h. No differences between CHOW and CONT rats were detected at either time point. Similar results were observed on the first and second intermittent access to HFD on days 6 and 10, respectively. HFD intake on final binge day was increased in INT rats compared to CONT rats at (**B**) 30 min and (**E**) 2 h. Chow intake was increased in CHOW rats compared to CONT and INT rats at (**C**) 30 min and (**F**) 2 h on the final binge day, with no differences in chow intake seen between CONT and INT at either time point. * Group difference, *p* < 0.0001.

On the last INT HFD day (day 14), group differences in food intake (chow plus HFD) were detected within 30 min of access to the HFD, *F*(2, 37) = 22.89, *p* < 0.0001, η^2^ = 0.55 (Figure 3A), with total caloric intake increased in INT rats, relative to CHOW and CONT rats (*p*-values < 0.0001). The increase in 30 min food intake observed in INT rats was due to a selective increase in HFD consumption, with INT rats consuming about four times more HFD than CONT rats, *t*(26) = 5.53, *p* < 0.0001, d = −2.33 (Figure 3B). Group differences in 30 min chow intake were also detected, *F*(2, 37) = 52.71, *p* < 0.0001, η^2^ = 0.74 (Figure 3C), with CHOW rats consuming more than CONT and INT rats (*p*-values < 0.0001).

Similar results were observed at 2 h following access to HFD. INT rats consumed more total calories (chow plus HFD) than CHOW and CONT rats, *F*(2, 37) = 32.07, *p* < 0.0001, η^2^ = 0.63 (Figure 3D; *p*-values < 0.0001), and more HFD calories than CONT rats, *t*(26) = 5.97, *p* < 0.0001, d = 2.33 (Figure 3E). CHOW rats consumed more chow than CONT and INT rats at 2 h, *F*(2, 37) = 90.71, *p* < 0.0001, η^2^ = 0.83 (Figure 3F; *p*-values < 0.0001).

### 3.3. Intermittent Access to HFD Affected Microglial Number and Morphology in ARC

**Figure 4:**
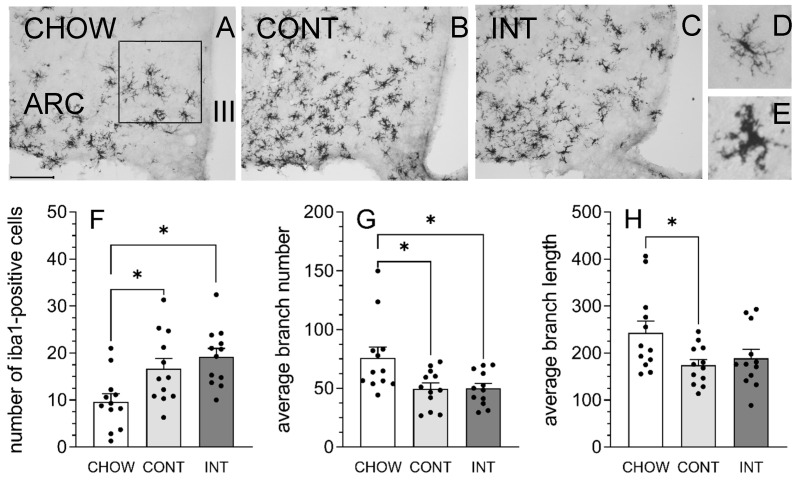
Both continuous and intermittent consumption of HFD promoted microglial reactivity in the ARC. (**A**–**C**) Representative images of Iba1-positive cells in CHOW, CONT, and INT rats (scale bar is 100 μm). The box represents the area of quantification within the ARC. Representative images of (**D**) a highly branched homeostatic microglial cell in a CHOW rat and (**E**) a reactive microglial cell in an INT rat. HFD consumption induced microglial reactivity in the ARC of CONT and INT rats compared to CHOW rats, as shown by the (**F**) increased number and (**G**) decreased average branch number of Iba1-positive cells. (**H**) Average branch length of Iba1-positive cells was decreased in CONT rats compared to CHOW rats. Expression of Iba1-positive cells (number and morphology) did not differ between CONT and INT rats. * Different from CHOW, *p* < 0.05.

Representative images of Iba1-positive cells within the ARC across the three diet groups are shown in Figure 4A through Figure 4C. Representative images of a homeostatic microglial cell (i.e., smaller cell body with long, skinny branches) and a microglial cell that appeared reactive to HFD consumption (i.e., larger cell body with retracted and thickened branches) are shown in Figure 4D,E. Diet condition influenced the number and morphology of Iba1-positive cells in the ARC, *F*(2, 33) = 3.58–6.88, *p*-values < 0.05–0.005, η^2^ = 0.18–0.29. Post hoc analyses revealed that CONT and INT rats had a greater number of Iba1-positive cells, and a decrease in the average branch number of Iba1-positive cells, relative to CHOW rats (*p*-values < 0.05) (Figure 4F,G). The average branch length of Iba1-positive cells was also decreased in CONT rats, relative to CHOW rats (*p* < 0.05) (Figure 4H). No differences in the number or morphology of Iba1-positive cells were detected between CONT and INT rats.

### 3.4. Intermittent Access to HFD Affected Microglial Morphology in NTS

**Figure 5:**
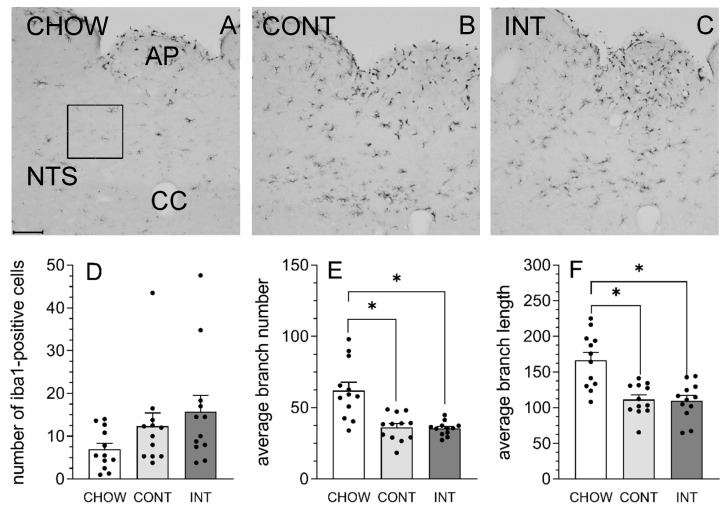
Both continuous and intermittent consumption of HFD promoted microglial reactivity in the NTS. (**A**–**C**) Representative images of Iba1-positive cells in the NTS in CHOW, CONT, and INT rats (scale bar is 100 μm). The box represents the area of quantification within the NTS. (**D**) There was a trend for an increase in the number of Iba1-positive cells in INT and CONT rats, compared to CHOW rats, but this failed to reach statistical significance. The average branch number (**E**) and branch length (**F**) of Iba1-positive cells was decreased in CONT and INT rats compared to CHOW rats. The expression of Iba1-positive cells (number and morphology) did not differ between CONT and INT rats. * Different from CHOW, *p* < 0.0005–0.0001.

Representative images of Iba1-positive cells within the NTS across the three diet groups are shown in Figure 5A through Figure 5C. While there was a trend for an increase in the number of Iba1-positive cells in CONT and INT rats compared to CHOW rats, these differences failed to reach statistical significance, *F*(2, 33) = 2.32, *p* = 0.114 (Figure 5D). Diet condition influenced microglial morphology, *F*(2, 33) = 15.87 and 14.21, *p*-values < 0.0001, η^2^ = 0.49 and 0.46, respectively, with decreases in the average branch number and branch length of Iba1-positive cells in CONT and INT rats, relative to CHOW rats (*p*-values < 0.001) (Figure 5E,F). No differences in the number or morphology of Iba1-positive cells were detected between CONT and INT rats.

### 3.5. Body Weight Gain and ARC Iba1 Expression in INT Rats Were Positively Correlated

**Figure 6:**
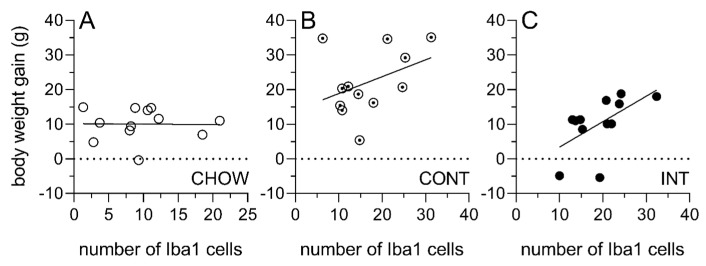
Correlational analysis of relationship between weight gain and number of ARC Iba1-positive cells. There was no association between body weight gain and number of ARC Iba1-positive cells in CHOW rats ((**A**) individual data points denoted by open circles) or CONT rats ((**B**) individual data points denoted by open circles with a dot in the middle). In INT rats ((**C**) individual data points denoted by closed circles), weight gain was positively correlated with number of Iba1-positive cells, *p* = 0.05.

Correlations were performed to investigate the relationship between cumulative weight gain and the number of Iba1-positive cells in the ARC within each diet group. No association was detected between weight gain and the number of Iba1-positive cells in the ARC of CHOW rats, *r*(11) = −0.02, n.s. (Figure 6A). While there was a trend for the number of Iba1-positive cells to increase as a function of weight gain in CONT rats, the correlation failed to reach statistical significance, *r*(11) = 0.39, n.s. (Figure 6B). In comparison, weight gain was positively correlated with the number of Iba1-positive cells in the ARC of INT rats, *r*(11) = 0.58, *p* = 0.05 (Figure 6C).

## 4. Discussion

Intermittent access to HFD promoted overconsumption in female rats, relative to control rats consuming chow or chow plus HFD daily. This binge-like overconsumption occurred during the first 2 h of intermittent HFD access, when INT rats consumed almost four times more calories than the control rats. Following episodes of binge-like eating, INT rats displayed a compensatory decrease in chow intake that prevented the diet-induced excess weight gain observed in CONT rats consuming HFD daily. Despite consuming fewer total calories and about 50% fewer fat calories than CONT rats over the 14-day paradigm, microglial reactivity in the ARC and NTS was similar in INT and CONT rats. Taken together, our findings demonstrate that dysregulated (binge-like) eating promotes microglial reactivity in brain areas that control food intake, and that this immune response is not related to excess weight gain.

As is consistent with previous studies using the intermittent HFD rodent model, we found that INT rats overconsumed calories on days of HFD access, relative to CHOW and CONT rats [24,34,35]. This overconsumption of calories was evident within the first 30 min of HFD access, as is consistent with a previous study in which the size of the first meal on intermittent HFD days was increased in INT rats, relative to CHOW and CONT rats [35]. This group also reported a blunted anorexigenic response to the intragastric infusion of nutrients and exogenous administration of amylin, a meal-related satiation signal, in INT rats relative to CHOW and CONT rats [35]. These findings suggest that an impairment in meal-related satiation signals contributes to the overconsumption of calories observed in INT rats when HFD is available. Here, we found that INT rats consumed fewer calories than CONT and CHOW rats on days following HFD access, and this compensatory hypophagia prevented the diet-induced increases in total food intake, HFD intake, and weight gain observed in CONT rats. While INT rats appear to display meal-related deficits in satiation when HFD is available, the compensatory hypophagia and lack of excess weight gain suggest that they remain sensitive to meal-related satiation signals when returned to a chow diet, at least over the course of the current study. Our finding that INT rats gained no more weight than CHOW rats further suggests that INT rats remained sensitive to longer-term adiposity signals, such as leptin, that regulate energy balance.

Rodent models of diet-induced obesity have demonstrated that chronic, unrestricted access to HFD promotes neuroinflammation in the mediobasal hypothalamus, with the ARC being particularly vulnerable [16,18,19,36,37,38]. We extend this literature by demonstrating that intermittent overconsumption of HFD, during three discrete episodes of binge-like eating, promotes the accumulation of microglia in the ARC of INT rats, as quantified by an increase in the number of Iba1-positive cells. Further analysis of these microglial cells revealed shortened, thickened processes and decreased branching, as is consistent with the morphological changes seen in microglia responding to physiological immune threats, including the acute administration of lipopolysaccharide [39]. While it will be important in future studies to examine correlates of neuroinflammation, including proinflammatory cytokines and the activity in downstream inflammatory signaling pathways, these morphological changes suggest that microglia were responding to the increase in fatty acids occurring during discrete episodes of binge-like eating. Indeed, the ARC is ideally positioned to detect and respond to excess fatty acids and other peripheral metabolic signals given its proximity to the median eminence, a circumventricular organ with fenestrated capillaries [16]. Interestingly, the pattern of microglial reactivity in the ARC was similar in INT and CONT rats, even though INT rats consumed about four-fold fewer fat calories than CONT rats across our study. To the best of our knowledge, this provides the first evidence that intermittent overconsumption of HFD, specifically three discrete episodes of binge-like eating, can promote microglial reactivity in the ARC.

The ARC contains two populations of neurons that play critical roles in regulating energy homeostasis. The activation of anorexigenic proopiomelanocortin (POMC) neurons reduces food intake and increases energy expenditure, whereas the activation of orexigenic agouti-related protein/neuropeptide Y (AgRP/NPY) neurons exert the opposite effects on food intake and energy expenditure. Interestingly, microglia reacting to prolonged HFD consumption accumulate in closer proximity to POMC neurons than AgRP/NPY neurons [40], and one study demonstrated that the persistent neuroinflammation and microglial reactivity observed following 8 months of HFD consumption in mice was associated with a selective decrease in POMC neurons [19]. Whether the microglial reactivity observed in our CONT and INT rats had a similar deleterious effect on POMC neurons is unknown, but it is possible based on other studies showing that microglia can promote neuronal injury and synaptic pruning/reorganization within one week of HFD consumption [21,22].

Intermittent overconsumption of HFD was also associated with an increase in microglial numbers and changes in microglial morphology, suggesting a reactive state in the NTS of INT rats, but only the latter was statistically reliable. As was observed in the ARC, microglial reactivity in the NTS was similar in INT and CONT rats. Our findings extend previous studies showing that chronic, unrestricted access to HFD can induce microglial reactivity in the NTS of rodents [18,38,41,42,43] by demonstrating that three days of intermittent overconsumption of HFD, occurring in discrete episodes of binge-like eating, promotes a microglial response in the NTS that is like that observed in chronically HFD-fed rats. In future studies, it will be important to examine the relationship between chronic binge eating and neuroinflammation and determine whether neuroinflammation persists in animals with a history of engaging in binge eating.

The NTS was examined given the critical role it plays in controlling meal size [26,27]. Because binge eating spectrum disorders are defined by objectively large meals, and the intermittent availability of HFD promotes large (binge-like) meals in rodents as seen here and in previous studies [24,35], the NTS represents a likely site of dysregulation in binge eating. Additionally, meal-related satiation signals, which may be disrupted in binge eating spectrum disorders [35], are relayed to the brain via vagal afferent neurons that terminate in the NTS [38], and HFD consumption was shown to alter this vagal gut–brain communication. Specifically, four weeks of HFD consumption promoted microglial reactivity in the nodose ganglion of rats and this was accompanied by withdrawal of vagal innervation in the gut and NTS [41]. Future studies are needed to assess whether an inflammation-dependent disruption in vagal input to the NTS may contribute to the overconsumption of calories in rats given intermittent access to HFD.

The compensatory hypophagia observed in INT rats on days following HFD access prevented the diet-induced increase in total food intake and obesity phenotype observed in CONT rats. As such, the microglial reactivity observed in the ARC and NTS of INT rats may have been driven by dysregulated (binge-like) eating behavior, rather than diet-induced weight gain. These findings in the ARC are consistent with a prior study in which hypothalamic microglial reactivity appeared to be induced by HFD independent of weight gain in mice [44]. Our findings also support prior work in humans linking binge eating to inflammation, independent of body weight. For example, our group has shown that binge eating is uniquely associated with increased plasma IL-6, a proinflammatory cytokine, in a transdiagnostic group of individuals with binge eating spectrum disorders and body mass indices ranging from underweight to obese [12]. Others have shown that a diagnosis of binge eating disorder in individuals with obesity is associated with increased markers of peripheral inflammation, including a poorer metabolic profile and lower levels of adiponectin, in comparison to individuals with obesity who have not been diagnosed with an eating disorder [13,14]. Our findings of increased microglial reactivity in the absence of an obesity phenotype in INT rats is also consistent with rodent studies in which unrestricted access to HFD was shown to increase microglial reactivity in the brain within days and prior to significant weight gain [16,18,19,45]. Based on these studies, it was hypothesized that the early microglial response to HFD consumption may be protective, whereas the sustained microglial reactivity observed in rodents with obesity may have detrimental effects, including the loss of POMC neurons in the ARC [19]. The rodent model of intermittent versus chronic HFD access used here provides a unique opportunity to further dissect the effects of dysregulated eating behavior versus diet-induced weight gain on neuroinflammation. In this regard, future studies comparing peripheral and central markers of inflammation in INT rats following one versus multiple episodes of binge-like eating in comparison to CONT rats with unrestricted access to HFD may help clarify how changes in microglial reactivity may promote or maintain dysregulated eating behavior and diet-induced obesity.

Previously, microglial accumulation in the ARC was positively correlated with body weight in obese rats fed a HFD for 8 weeks [19]. In the current study, we found a positive association between the number of ARC microglia and body weight in CONT, and to a greater extent in INT rats, with no association in CHOW rats. Taken together, our findings suggest that this positive correlation between microglial accumulation in the ARC and body weight is dependent on the availability of HFD and that it may be driven by dysregulated eating behavior, given the stronger association in INT rats that engaged in binge-like eating, relative to CONT rats. Thus, while the increase in microglial reactivity observed in INT rats was not dependent on the development of an obesity phenotype, individual differences in the accumulation of microglia in the ARC did predict weight gain within this group.

Here, we provide the first demonstration that intermittent overconsumption of HFD, occurring in discrete (2 h) episodes of binge-like eating, promotes microglial reactivity in the ARC and NTS of female rats. Because INT rats failed to develop the obesity phenotype observed in CONT rats given unrestricted access to HFD, the changes in microglial reactivity in INT rats appear to be driven by dysregulated (binge-like) eating behavior, rather than excess weight gain/adiposity. The accumulation of microglia in the ARC of INT rats was, however, positively correlated with body weight. Taken together, these findings support a model in which neuroinflammation, assessed via increased microglial reactivity in brain areas that control food intake, may promote the overconsumption of calories and thereby maintain or exacerbate binge-like eating in this rodent model. In light of the clinical research demonstrating an association between inflammation and binge eating spectrum disorders, the current findings have important implications in terms of increasing our understanding of the neuroimmune factors that contribute to binge eating. Our findings are limited, however, by the caveat that rodent models of binge eating cannot recapitulate the full spectrum of complex symptoms expressed in individuals with binge eating spectrum disorders, and this constrains the degree to which our findings can be broadly translated to clinical populations. Additional studies are also needed to determine whether our findings in females extend to males. Nevertheless, our current findings associating intermittent binge-like eating with neuroinflammation provide new insights into the role of the immune system in binge eating spectrum disorders while also identifying biological targets that could lead to the development of novel, more efficacious treatments for reducing binge eating.

## Data Availability

All data are presented in this manuscript.

## Abbreviations

AGRP, agouti-related protein; ANOVA, analysis of variance test; AP, area postrema; ARC, arcuate nucleus of the hypothalamus; BED, binge eating disorder; BN, bulimia nervosa; CC; central canal; CHOW, chow diet; CONT, continuous high fat diet; HFD, high fat diet; Iba1, ionized calcium-binding adaptor molecule 1; III, third ventricle; INT, intermittent high fat diet; NPY, neuropeptide Y; NTS, nucleus of the solitary tract; POMC, proopiomelanocortin; ROI, region of interest.

## References

[1] Mathes W.F., Brownley K.A., Mo X., Bulik C.M. (2009). The Biology of Binge Eating. Appetite.

[2] Breton E., Fotso Soh J., Booij L. (2022). Immunoinflammatory Processes: Overlapping Mechanisms Between Obesity and Eating Disorders?. Neurosci. Biobehav. Rev..

[3] Meng Y., Kautz A. (2022). An Evidence Review of the Association of Immune and Inflammatory Markers with Obesity-Related Eating Behaviors. Front. Immunol..

[4] Raevuori A., Haukka J., Vaarala O., Suvisaari J.M., Gissler M., Grainger M., Linna M.S., Suokas J.T. (2014). The Increased Risk for Autoimmune Diseases in Patients with Eating Disorders. PLoS ONE.

[5] Zerwas S., Larsen J.T., Petersen L., Thornton L.M., Quaranta M., Koch S.V., Pisetsky D., Mortensen P.B., Bulik C.M. (2017). Eating Disorders, Autoimmune, and Autoinflammatory Disease. Pediatrics.

[6] Solmi F., Bulik C.M., De Stavola B.L., Dalman C., Khandaker G.M., Lewis G. (2020). Longitudinal Associations between Circulating Interleukin-6 and C-Reactive Protein in Childhood, and Eating Disorders and Disordered Eating in Adolescence. Brain Behav. Immun..

[7] Caroleo M., Carbone E.A., Greco M., Corigliano D.M., Arcidiacono B., Fazia G., Rania M., Aloi M., Gallelli L., Segura-Garcia C. (2019). Brain-Behavior-Immune Interaction: Serum Cytokines and Growth Factors in Patients with Eating Disorders at Extremes of the Body Mass Index (Bmi) Spectrum. Nutrients.

[8] Corcos M., Guilbaud O., Paterniti S., Moussa M., Chambry J., Chaouat G., Consoli S.M., Jeammet P. (2003). Involvement of Cytokines in Eating Disorders: A Critical Review of the Human Literature. Psychoneuroendocrinology.

[9] Dalton B., Bartholdy S., Robinson L., Solmi M., Ibrahim M.A.A., Breen G., Schmidt U., Himmerich H. (2018). A Meta-Analysis of Cytokine Concentrations in Eating Disorders. J. Psychiatr. Res..

[10] Tabasi M., Anbara T., Siadat S.D., Khezerloo J.K., Elyasinia F., Bayanolhagh S., Safavi S.A.S., Yazdannasab M.R., Soroush A., Bouzari S. (2020). Socio-Demographic Characteristics, Biochemical and Cytokine Levels in Bulimia Nervosa Candidates for Sleeve Gastrectomy. Arch. Iran. Med..

[11] Raymond N.C., Dysken M., Bettin K., Eckert E.D., Crow S.J., Markus K., Pomeroy C. (2000). Cytokine Production in Patients with Anorexia Nervosa, Bulimia Nervosa, and Obesity. Int. J. Eat. Disord..

[12] Campanile A.A., Eckel L.A., Keel P.K. (2024). Elevated Interleukin-6 in Women with Binge-Eating Spectrum Disorders. Int. J. Eat. Disord..

[13] Brandão P.P., Garcia-souza E.P., Neves F.A., Jose M., Sichieri R., Moura E.G.D., Cristina P., Moura A.S. (2010). Leptin/Adiponectin Ration in Obese Women with and without Binge Eating Disorder. Neuroendocrinol. Lett..

[14] Succurro E., Segura-Garcia C., Ruffo M., Caroleo M., Rania M., Aloi M., De Fazio P., Sesti G., Arturi F. (2015). Obese Patients with a Binge Eating Disorder Have an Unfavorable Metabolic and Inflammatory Profile. Medicine.

[15] Dorfman M.D., Thaler J.P. (2015). Hypothalamic Inflammation and Gliosis in Obesity. Curr. Opin. Endocrinol. Diabetes.

[16] Valdearcos M., Robblee M.M., Benjamin D.I., Nomura D.K., Xu A.W., Koliwad S.K. (2014). Microglia Dictate the Impact of Saturated Fat Consumption on Hypothalamic Inflammation and Neuronal Function. Cell Rep..

[17] André C., Quevedo O.G., Rey C., Rémus-Borel J., Clark S., Castellanos-Jankiewicz A., Ladeveze E., Leste-Lasserre T., Nadjar A., Abrous D.N. (2017). Inhibiting Microglia Expansion Prevents Diet-Induced Hypothalamic and Peripheral Inflammation. Diabetes.

[18] Butler M.J., Perrini A.A., Eckel L.A. (2020). Estradiol Treatment Attenuates High Fat Diet-Induced Microgliosis in Ovariectomized Rats. Horm. Behav..

[19] Thaler J.P., Yi C.-X., Schur E.A., Guyenet S.J., Hwang B.H., Dietrich M.O., Zhao X., Sarruf D.A., Izgur V., Maravilla K.R. (2012). Obesity Is Associated with Hypothalamic Injury in Rodents and Humans. J. Clin. Investig..

[20] Graeber M.B., Streit W.J. (2010). Microglia: Biology and Pathology. Acta Neuropathol..

[21] Horvath T.L., Sarman B., García-Cáceres C., Enriori P.J., Sotonyi P., Shanabrough M., Borok E., Argente J., Chowen J.A., Perez-Tilve D. (2010). Synaptic Input Organization of the Melanocortin System Predicts Diet-Induced Hypothalamic Reactive Gliosis and Obesity. Proc. Natl. Acad. Sci. USA.

[22] Wang X.L., Li L. (2021). Microglia Regulate Neuronal Circuits in Homeostatic and High-Fat Diet-Induced Inflammatory Conditions. Front. Cell. Neurosci..

[23] Wang X., Li H. (2022). Chronic High-Fat Diet Induces Overeating and Impairs Synaptic Transmission in Feeding-Related Brain Regions. Front. Mol. Neurosci..

[24] Czyzyk T.A., Sahr A.E., Statnick M.A. (2010). A Model of Binge-like Eating Behavior in Mice That Does Not Require Food Deprivation or Stress. Obesity.

[25] Ullah R., Rauf N., Nabi G., Yi S., Yu-Dong Z., Fu J. (2021). Mechanistic Insight into High-Fat Diet-Induced Metabolic Inflammation in the Arcuate Nucleus of the Hypothalamus. Biomed. Pharmacother..

[26] Grill H.J. (2011). Leptin and the Systems Neuroscience of Meal Size Control. Front. Neuroendocrinol..

[27] Smith G.P. (1996). The Direct and Indirect Controls of Meal Size. Neurosci. Biobehav. Rev..

[28] Ito D., Imai Y., Ohsawa K., Nakajima K., Fukuuchi Y., Kohsaka S. (1998). Microglia-Specific Localisation of a Novel Calcium Binding Protein, Iba1. Mol. Brain Res..

[29] National Research Council (US) Committee for the Update of the Guide for the Care and Use of Laboratory Animals (2011). Guide for the Care and Use of Laboratory Animals.

[30] Percie du Sert N., Hurst V., Ahluwalia A., Alam S., Avey M.T., Baker M., Browne W.J., Clark A., Cuthill I.C., Dirnagl U. (2020). The ARRIVE Guidelines 2.0: Updated Guidelines for Reporting Animal Research. J. Cereb. Blood Flow Metab..

[31] Spencer S.J., Basri B., Sominsky L., Soch A., Ayala M.T., Reineck P., Gibson B.C., Barrientos R.M. (2019). High Fat Diet Worsens the Impact of Ageing on Microglial Function and Morphology in a Region-Specific Manner. Neurobiol. Aging.

[32] Paolicelli R., Sierra A., Stevens B., Tremblay M.-E., Aguzzi A., Ajami B., Amit I., Audinat E., Bechmann I., Bennett M. (2022). Defining Microglial States and Nomenclature: A Roadmap to 2030. Neuron.

[33] Young K., Morrison H. (2018). Quantifying Microglia Morphology from Photomicrographs of Immunohistochemistry Prepared Tissue Using Imagej. J. Vis. Exp..

[34] Bello N.T., Guarda A.S., Terrillion C.E., Redgrave G.W., Coughlin J.W., Moran T.H. (2009). Repeated Binge Access to a Palatable Food Alters Feeding Behavior, Hormone Profile, and Hindbrain c-Fos Responses to a Test Meal in Adult Male Rats. Am. J. Physiol. Integr. Comp. Physiol..

[35] Maske C.B., Coiduras I.I., Ondriezek Z.E., Terrill S.J., Williams D.L. (2020). Intermittent High-Fat Diet Intake Reduces Sensitivity to Intragastric Nutrient Infusion and Exogenous Amylin in Female Rats. Obesity.

[36] Milanski M., Degasperi G., Coope A., Morari J., Denis R., Cintra D.E., Tsukumo D.M.L., Anhe G., Amaral M.E., Takahashi H.K. (2009). Saturated Fatty Acids Produce an Inflammatory Response Predominantly through the Activation of TLR4 Signaling in Hypothalamus: Implications for the Pathogenesis of Obesity. J. Neurosci..

[37] Miller A., Spencer S. (2014). Obesity and Neuroinflammation: A Pathway to Cognitive Impairment. Brain Behav. Immun..

[38] Vaughn A.C., Cooper E.M., Dilorenzo P.M., Loughlin L.J.O., Konkel M.E., Peters J.H., Hajnal A., Sen T., Lee S.H. (2017). Energy-Dense Diet Triggers Changes in Gut Microbiota, Reorganization of Gut-Brain Vagal Communication and Increases Body Fat Accumulation. Acta Neurobiol. Exp..

[39] Frank M.G., Fonken L.K., Watkins L.R., Maier S.F. (2020). Microglia: Neuroimmune-Sensors of Stress. Semin. Cell Dev. Biol..

[40] Gao Y., Bielohuby M., Fleming T., Grabner G.F., Foppen E., Bernhard W., Guzmán-Ruiz M., Layritz C., Legutko B., Zinser E. (2017). Dietary Sugars, Not Lipids, Drive Hypothalamic Inflammation. Mol. Metab..

[41] Sen T., Cawthon C.R., Ihde B.T., Hajnal A., Dilorenzo P.M., De C.B., Serre L., Czaja K., Barbier C., Author P.B. (2017). Diet-Driven Microbiota Dysbiosis Is Associated with Vagal Remodeling and Obesity HHS Public Access Author Manuscript. Physiol. Behav..

[42] Minaya D.M., Turlej A., Joshi A., Nagy T., Weinstein N., DiLorenzo P., Hajnal A., Czaja K. (2020). Consumption of a High Energy Density Diet Triggers Microbiota Dysbiosis, Hepatic Lipidosis, and Microglia Activation in the Nucleus of the Solitary Tract in Rats. Nutr. Diabetes.

[43] Kim J.S., Kirkland R.A., Lee S.H., Cawthon C.R., Rzepka K.W., Minaya D.M., de Lartigue G., Czaja K., de La Serre C.B. (2020). Gut Microbiota Composition Modulates Inflammation and Structure of the Vagal Afferent Pathway. Physiol. Behav..

[44] Gao Y., Ottaway N., Schriever S.C., Legutko B., García-Cáceres C., de la Fuente E., Mergen C., Bour S., Thaler J.P., Seeley R.J. (2014). Hormones and Diet, but Not Body Weight, Control Hypothalamic Microglial Activity. Glia.

[45] Ávalos Y., Kerr B., Maliqueo M., Dorfman M. (2018). Cell and Molecular Mechanisms behind Diet-Induced Hypothalamic Inflammation and Obesity. J. Neuroendocrinol..

